# Characteristics and predictors for hospitalizations of home-dwelling older persons receiving community care: a cohort study from Norway

**DOI:** 10.1186/s12877-018-0887-z

**Published:** 2018-09-03

**Authors:** Martha Therese Gjestsen, Kolbjørn Brønnick, Ingelin Testad

**Affiliations:** 1 Centre for age-related medicine (SESAM), Stavanger University Hospital, Stavanger, Norway; 2 University of Stavanger, Faculty of Health Sciences, Centre for Resilience in Healthcare (SHARE), Stavanger, Norway; 3 Centre for Clinical Research in Psychosis (TIPS), Stavanger University Hospital, Stavanger, Norway; 4 University of Stavanger, Faculty of Health Sciences, Stavanger, Norway; 5 University of Exeter Medical School, Exeter, Devon UK

**Keywords:** Hospitalizations, Elderly, Community care, Assistive living technology, Prevention

## Abstract

**Background:**

Older persons are substantial consumers of both hospital- and community care, and there are discussions regarding the potential for preventing hospitalizations through high quality community care. The present study report prevalence and factors associated with admissions to hospital for community-dwelling older persons (> 67 years of age), receiving community care in a Norwegian municipality.

**Methods:**

This was a cohort study of 1531 home-dwelling persons aged ≥67 years, receiving community care. We retrospectively scrutinized admissions to hospital for the study cohort over a one-year period in 2013. The frequency of admissions was evaluated with regard to association with age (age groups 67–79 years, 80–89 years and ≥ 90 year) and gender. The hospital admission incidence was calculated by dividing the number of admissions by the number of individuals included in the study cohort, stratified by age and gender. The association between age and gender as potential predictors and hospitalization (outcome) was first examined in univariate analyses followed by multinomial regression analyses in order to investigate the associations between age and gender with different causes of hospitalization.

**Results:**

We identified a total of 1457 admissions, represented by 739 unique individuals, of which 64% were women, and an estimated mean age of 83 years. Mean admission rate was 2 admissions per person-year (95% confidence interval (CI): 1.89–2.11). The admission rate varied with age, and hospital incidents rates were higher for men in all age groups. The overall median length of stay was 4 days. The most common reason for hospitalization was the need for further medical assessment (23%). We found associations between increasing age and hospitalizations due to physical general decline, and associations between male gender and hospitalizations due to infections (e.g., airways infections, urinary tract infections).

**Conclusions:**

We found the main reasons for hospitalizations to be related to falls, infections and general decline/pain/unspecified dyspnea. Men were especially at risk for hospitalization as they age. Our study have identified some clinically relevant factors that are vital in understanding what health care personnel in community care need to be especially aware of in order to prevent hospitalizations for this population.

## Background

The global phenomena of ageing populations (> 65 years) [1] alongside reduced number of personnel available for both formal and informal care [2], may threaten the sustainability of the health care systems [3, 4]. Persons over 65 years of age are substantial consumers of both hospital- and primary care [5, 6], and a peak in hospitalization rates for both men and women can be seen in all European countries through the age group 80 and over [1]. In Norway, 35% of all individuals over 80 years were hospitalized in 2013 and 68% of these also received community health- and care services [6]. Also, older persons over 65 years accounted for nearly 27% of all overnight stays, while only comprising 11% of the population, and an increase in over-night stays from 17.8% in 2003, to 19.6% in 2013 were shown within this population [5]. The proportion of increasing age is thus associated with an increasing demand for specialized health care [7, 8], and this rising demand for acute hospital beds leads to a strong policy interest in identifying interventions which are effective in reducing avoidable hospital admissions [9–13].

Previous studies on factors predicting hospitalizations of older persons have reported different findings, but there is a discrepancy in findings regarding risk factors associated with hospitalizations for older persons. Whereas quite a few studies have found that a previous hospital admission were associated with a higher risk to be re-hospitalized [12, 14, 15], Roland and colleagues [16] found that having two or more admissions one year, proved to have a low sensitivity in detecting older patients who will have high admissions in the following year. Several studies underline that the severity of disease and the burden of comorbidity are strong predictors of hospitalizations [11, 12, 15, 17], and also that functional disability, cognitive impairment, as well as factors related to living conditions (i.e., low socio-economic level and social deprivation) also seem to play a part in frequency of hospitalizations for older persons [15, 18, 19].

Gender differences in health care utilization are illustrated in several studies, but are inconclusive as to whether being male or female is a risk factor [12]. Some studies found that men above the age of 80 had approximately 25% more inpatient stays than women in the same age group [20–22] but others find that female sexis associated with multi-morbidity, and consequently have an increased risk of hospitalization [23].

A literature review [24] identified nine predictors which were independently associated with unplanned admissions to hospital in older people aged over 75 years: male gender, history of falls in the previous 12 months, ischaemic heart disease, respiratory disease, atrial fibrillation, cancer, having leg ulceration, living alone without help and having difficulty with mobility. Other studies have identified that emergency hospital admissions often occurs when an older person has reached a point of crisis, due to a combination of circumstances; such as an exacerbation of a chronic condition, change in social setting, or a cascade of symptoms due to multi-morbidity and frailty [12, 17, 25, 26].

The various risk factors related to hospitalizations for older persons, as identified in previous research, are summed up in Table 1.

**Table 1:** Various risk factors associated with hospitalizations for older persons

Risk domain	Specific risk factors
Age	Increasing age
Frequency of hospitalizations	Previous hospitalization
Gender	Male Female
Health-related conditions	Severity of disease Comorbidity Functional decline/disability Respiratory disease Ischaemic heart disease Atrial fibrillation Cancer Leg ulcers
Living conditions	Low socio-economic level Deprivation Living alone, without help
Behavioral factors	Lack of exercise Falls Poor nutrition

It is an ongoing discussion whether a proportion of the hospital admissions among older persons could have been prevented in primary treatment and care [7, 27, 28]. Studies from Scandinavia have found that older persons are hospitalized due to lack of an appropriate alternative in primary care [28, 29], however a Norwegian study found no association between the volume of municipality general practitioners provided (in a universally accessible healthcare system) and unplanned hospitalizations of the entire elderly population (aged ≥65 years) [8, 30]. The picture concerning the prevention of hospitalizations within this age group is thus not clear; heterogeneity in terms of health status and age-related conditions, as well as numerous contextual factors related to the health care system, represent a challenge for isolating factors concerning thematter. It is therefore of vital importance to understand the actual clinical reasons for hospitalization in order to develop more timely and appropriate care services interventions [9, 15, 31], as well as the impact of policy efforts to reduce and prevent avoidable hospitalizations [10].

Part of the policy efforts is to shift resources from hospitals to the community care setting, and in this context the use of assistive living technologies is suggested to help monitor and treat degenerative and chronic diseases through the use of sensors, alarms and reminders [32, 33]. In a review by Purdy & Huntley [27], the use of automated vital signs monitoring and telephone follow-up by nurses was promising with regards to preventing and reducing avoidable emergency admissions.

Previous research underlines that more studies are needed to assess outcome and effectiveness related to the use of assistive living technologies in the context of preventing hospitalizations for older persons [34, 35], but there is a potential to do so by providing early warnings of exacerbation events or deterioration. This is a significant issue in regard to both quality and cost [24, 32].

This knowledge can further contribute to develop appropriate assistive living technology interventions, thus focussing on timely interventions in primary care together with understanding the actual clinical reasons for hospitalization.

Therefore, to identify ways to prevent hospitalizations with the use of assistive living technology, the aim of this study is to identify the reason for referral to hospital, and further to describe the prevalence and correlates associated with admissions to hospital for home-dwelling older persons (> 67 years of age) receiving community care in a Norwegian municipality.

More specifically, we will

i. Describe the frequency related to reasons for referral, and characteristics of hospital admissions of home-dwelling older persons receiving community care.
ii. Describe the associations between demographic characteristics and admission to hospital.

## Methods

### Study design and setting

This is a descriptive, cohort study of 1531 home-dwelling persons aged ≥67 years, receiving community-based care in a Norwegian municipality. Demographic characterisitics of the study cohort are presented in Table 2. According to the World Health Organization (WHO), the age cut-off is 60+ years to refer to the older or elderly persons [36]. This study however, has applied the age-cutoff as provided by Statistics Norway, because when extracting information about health service provision in Norway, 67 years of age is the standard age-distinction. The study was carried out in a municipality where 10.4% of the population is ≥67 years of age [37]. The number of cases in this cohort was determined by the number of hospitalizations during the one-year study period and thus, they are mirroring the influence of ageing on hospital admissions, as they closely match the current age structure of the Norwegian population receiving community care [38]. We retrospectively scrutinized admissions to hospital for the study cohort between April 1st 2012 and March 31st 2013. Data were collected electronically from existing registries. The studied hospitalizations stems from a hospital located in an urban area, it is the only hospital within an 80 km radius and serves approximately 365,000 persons.

**Table 2:** Demographic characteristics of study cohort

Selected variables	% of total (*N* = 1531)		Mean ± sd
*Gender*:			
Male	32.6		
Female	67.4		
*Age*:		*Male* *(% within age group)*	
67–79	27.1	40.7	
80–89	43.3	31.9	
90 +	29.6	24.9	
Mean age			83.7 ±
			7.435

*Sd* standard deviation

### Community care

Community care represents the lowest level of care services provided by the municipality and there are few formal demands required in order to receive community care in Norway. The proper instance in the health- and social district one geographically belongs to, defines the need for assistance and/or care, together with the person seeking help. Referrals to hospital are made either from patients’ general practitioner, or from an out-of-hours community-based emergency department. The persons included in our study received services from the municipality, including medical care provided by nurses (medication, wound/ulcer dressing, personal hygiene) and practical home care provided by formal carers (not necessarily nurses).

### Variables and data analysis

The variables entered into the analysis were selected primarily for their clinical importance, based on previous research to be essential [9, 39–41], and included gender, age and reason for referral. The primary reason for referral to hospital was retrieved through hospital-based patient records, based on the International Classification of Diseases version 10 (ICD-10) main chapters. When reason for referral to hospital was inexplicit (i. e., to clarify whether the patient was referred either for COPD exacerbation or pneumonia), the first author checked the patients’ hospital records to identify the most accurate reason for referral. A second rater evaluated the reasons for referral to hospital for 141 randomly selected cases, and then we performed an agreement-testing, using Cohen’s Kappa (*κ*) to test interrater reliability [31]. The coefficient was 0.7, which supports the reliability and validity of the rating procedure. Length of stay (LoS) was calculated from admission to discharge date and presented in days; for persons who had less than 6 hours at the hospital, LoS is calculated to be 0 days.

Continuous variables are described as means and standard deviations, while categorical variables are reported as frequencies.

The hospital admission incidence was calculated by dividing the number of admissions by the number of individuals included in the study cohort, stratified by age and gender (Table 3). Frequency of admissions was evaluated separately for each reason for referral for the age groups 67–79 years, 80–89 years and ≥ 90 year using Z-tests for testing differences of admission proportions in each age group for each reason for referral to hospital. Confidence intervals are also reported for each age group. A multinomial logistic regression analysis was then performed in order to investigate the partial, independent effects of age and gender on the most common reasons for referral to hospital (fall, infections or general decline) The dependent variable was categorical, i.e., fall, infections or general decline using no hospitalizations as a reference group. Age and gender were entered as predictor variables. Alpha level was set at *p* < .05. All statistical analyses were conducted using SPSS Release 23.0.0.0 (IBM, Inc., Chicago, IL, USA).

**Table 3:** Hospital incidence rate for the study cohort, stratified on gender

Total				Men			Women		
			Mean			Mean			Mean
Persons Total cohort		Admissions (*n*)	annual admission rate	Persons - age	Admissions *(n)*	annual admission rate	Persons - age	Admissions *(n)*	annual admission rate
1531		1457	0.95	494	566	1.1	1038	891	0.9
67–79 years	415	426	1.0	169	210	1.2	246	216	0.9
80–89 years	664	655	0.98	212	254	1.2	452	401	0.9
90+ years	453	377	0.83	113	102	0.9	340	274	0.8

## Results

### Demographic and frequencies related to hospitalizations

We identified a total of 1457 admissions, represented by 729 unique individuals from the study cohort (*n* = 1531), out of which 64% were women. The estimated mean age was 83 years. 384 persons (53%) of the hospitalized individuals (*n* = 729) were admitted only once during the study period. 169 individuals (23%) were admitted twice, 78 (11%) were admitted three times, while 98 persons (13%) were admitted more than four times during the one-year study period. The mean admission rate was 2 admissions per person-year (95% confidence interval (CI): 1.89–2.11). The overall median length of stay was 4 days (mean = 7.21, SD ± 9.9, range 1–138, interquartile range (IQR) =7). The most common reason for referral was the need for further medical assessment due to general decline, based on symptoms such as pain/unspecified dyspnea/dehydration/anemia (334 referrals = 23%). 303 referrals related to infections (ICD-10 chapter A J K L N) constituted nearly 21% of overall admissions, while falls caused 13% (191 referrals) of the hospitalizations for the study cohort. The most common reason for referral within infections were related to the respiratory system (e.g.*,* pneumonia), urinary tract infections and skin infections (e.g.*,* erysipelas). These results are depicted in Table 4. Some hospital admissions were associated with age, whilst others were associated with gender.

**Table 4:** Differences in age groups for different reasons for referral to hospital

Reason for referral		A: 67–79 years	B: 80–89 years	C: 90+ years	*p*-value*
	Frequency of admissions (%)	426 (29.2)	655 (45.0)	376 (25.8)	
Fall/accident	191 (13.1)	36 (8.5)	77 (11.8)	78 (20.7)	
Z-score (C.I.)		0.083 (0.062–0.115)	0.118 (0.095–0.144)	0.207 (0.169–0.251)	A vs B = .82; A vs C < .01; B vs C < .01
Infection	303 (20.8)	98 (23.0)	143 (21.8)	62 (16.5)	
Z-score (C.I.)		0.23 (0.193–0.272)	0.218 (0.188–0.252)	0.165 (0.131–0.206)	A vs B = .65; A vs C = .02; B vs C = .04
General decline/pain/unspecified dyspnea	334 (22.9)	65 (15.3)	159 (24.3)	110 (29.3)	
Z-score (C.I.)		0.153 (0.122–0.189)	0.243 (0.211–0.277)	0.293 (0.248–0.340)	A vs B < .01; A vs C < .01; B vs C = .08
Unspecified chest pain	90 (6.2)	29 (6.8)	41 (6.3)	20 (5.3)	
Z-score (C.I)		0.068 (0.048–0.096)	0.063 (0.046–0.084)	0.053 (0.035–0.081)	A vs B = .72; A vs C = .38; B vs C = .54
Heart attack	43 (3)	8 (1.9)	22 (3.4)	13 (3.5)	
Z-score		0.019 (0.009–0.037)	0.034 (0.022–0.050)	0.035 (0.020–0.058)	A vs B = .15; A vs C = .16; B vs C = .94
Congestive heart failure	58 (4)	15 (3.5)	25 (3.8)	18 (4.8)	
Psychiatry	41 (2.8)	38 (8.9)	2 (0.3)	1 (0.3)	
Old age psychiatry	32 (2.2)	7 (1.6)	16 (2.4)	9 (2.2)	
Neurology	91 (6.2)	20 (4.7)	47 (7.2)	24 (6.4)	
Cancer	137 (9.4)	68 (16.0)	56 (8.5)	13 (3.5)	
COPD	37 (2.5)	17 (4.0)	18 (2.7)	2 (0.5)	
GI symptoms	100 (6.9)	25 (5.8)	49 (7.5)	26 (6.9)	
Total (%)	1457 (100)	426 (29.2)	655 (45.0)	376 (25.8)	

### Age as a predictor for hospitalization

We found a higher admission rate in the lowest age group (67–79 years), compared to the other two age groups; the youngest had a mean admission rate of 1.0, which is slightly higher than the mean annual admission rate for the whole study population (.95) (see Table 3). I. e., the annual admission rate varied with age, but there was a statistically significant negative correlation between age and annual admission rate (Spearman’s rho = .-117, CI -.186- -.041, *p* = .002). We investigated this issue further by testing differences in proportions of hospitalizations in the three age groups related to the various reasons for referral to hospital using Z tests. We found that in connection to hospitalizations due to fall and infections, there was a statistically significant difference in proportions between the lowest and the highest age group (Fall *p* < .01; infections *p* = .02), and likewise between the middle age group and the highest age group (Fall *p* < .01; Infections *p* = .04), but not between the lowest and the middle age group (Fall *p* = .82; infections *p* = .65). As for general decline/pain/unspecified dyspnea as a reason for hospitalizations, we found a statistically significant difference in proportions between both the lowest and the middle age group (*p* < .01), as well as between the lowest and the highest age group (*p* < .01), but not between the middle and the highest age group (*p =* .08). The results are depicted in Table 4.

### Gender as a predictor for hospitalization

Overall, men had an annual admission rate of 1.1, while the corresponding rate for women was .9. The mean hospital admission rate for the entire study population was .95 (see Table 3). We found that hospital incidents rates were higher for men in all age groups, and further a statistically significant negative correlation between female gender and frequency of admission to hospital with a correlation coefficient (Spearman’s rho) of −.088 (CI 95%-.157- -.017, *p* = .018). This implies that in our study, being female was not associated with higher hospitalization rate, thus not presenting as a risk factor for admission to hospital.

The final prognostic index included age (categorized as 67–79, 80–89, ≥ 90), gender and reason for referral. We applied a multinomial logistic regression analysis to investigate whether age or gender were associated with admission to hospital (reason for referral) due to falls, infection or general decline (see Table 5). The results depicted in Table 5 shows the odds ratios for hospitalizations due to falls, infection or general decline vs. the reference group of no hospitalizations in the model.

**Table 5:** Predictors for hospitalization by multinomial logistic regression; demonstrating whether age or gender were associated with admission to hospital (reason for referral) due to falls, infection or general decline

Fall			Infection		General decline	
	OR (95% CI)	*P*-value	OR (95% CI)	P-value	OR (95% CI)	*P*-value
Age	1.03 (1.00–1.05)	.044	0.99 (0.97–1.02)	.469	1.04 (1.02–1.06)	**.001**
Gender	1.2 (0.77–1.86)	.418	0.47 (0.38–0.69)	**.000**	0.65 (0.45–0.93)	.017

*OR* Odds ratio, *CI* Confidence Interval. Alpha level 0.05. Bold values indicate variables that reached statistical significance. Reference group: No hospitalizations

Age was not a statistically significant predictor for hospitalization due to fall or infections, but we found that increasing age was associated with hospitalization due to general decline (*p* = .001). With regards to gender, we found that being male increased the odds for hospitalization when presenting symptoms related to infections by a factor of .5, being statistically significant (*p* < .001). As for associations between gender (=being male) and hospitalizations due to fall or general decline, the slightly increased odds were not statistically significant in either groups.

We further investigated whether there was a difference in the three age groups related to the various reasons for referral to hospital. In relation to hospitalization due to fall, we found a statistically significant difference (*p* = .01) between the youngest of age (age group A: 67–79 years) and the eldest (age group C: 90+), and also between the eldest (age group C) and age group B (80–89). There was no difference between age group A and B in this matter.

Also for hospitalizations due to an infection we found a statistically significance between the same age groups as for fall as reason for referral to hospital, i. e., between age groups A and C, and B and C.

Hospitalizations due to general decline had a slightly other expression; here we found a difference between age group A and B, and also between A and C, but not between B and C. The first and the latter result differ from the other two reasons for referral.

## Discussion

We found that 50% of the study cohort had at least one hospitalization during a one-year period, and that age and gender were associated with some hospitalizations. The most common reasons for referral were the need for further medical assessment, based on symptoms related to general decline, such as unspecified dyspnea/dehydration/anemia (23%), and referrals related to infections (21%) and falls (13%). More specifically we found that age was a predictor for hospitalization (*p*  ≤  .001) due to general decline, whereas in relation to falls and infections, we found no association between age and hospitalizations. We found that male gender was a predictor for hospitalizations due to infections (*P*  ≤  .000), but were not associated with hospitalizations related to falls or general decline. Several findings are noteworthy, especially in the context of current efforts using assistive living technologies to prevent hospitalizations for older persons.

First, the 50% admission rate we found highlight the point that this population is prone to conditions for which a doctor evaluates that a hospitalization is required. This is noteworthy in itself, but previous research have shown that taking only the frequency of admissions for older persons into account when predicting future admissions, have a low sensitivity [16]. We have therefore looked more into for which conditions older persons are hospitalized.

The most frequent reason for referral we identified in our study was general decline/pain/unspecified dyspnoea. This substantiate an already well-known perception that older persons often present general and diffuse symptoms before the doctor, and often may be in a severe state of illness [42]. Symptoms related to general decline/pain/unspecified dyspnoea could be related to non-communicable and chronic diseases, thus potentially preventable. However, these hospitalizations are often appropriate due to the degree of severity and the need for further assessment and examinations which only could be performed, in specialized health care [11, 15, 27]. The line of argument that follows the trajectory that high quality primary care prevents hospitalizations related to the reported symptoms, indicate that vigilant health care personnel in community care is a prerequisite for timely and accurate observations. The potential for preventing hospitalizations for this patient group lies in discovering and addressing the patients’ general decline and/or pain and/or unspecified dyspnea before the state of illness, where hospitalization is the only appropriate option for assessment, treatment and care. According to Fortinsky and colleagues [19], an increase on a dyspnea severity scale conferred an additional 18% greater likelihood of hospitalization, thus there could be a particular potential for preventing hospitalizations due to dyspnea symptoms. Moreover, monitoring such symptoms can be done through the use of assistive living technologies, as they allow a close and continuous monitoring of symptoms, systematic follow-up by health care personnel, and a proper response [43, 44].

The potential for preventing hospitalizations related to the second most frequent reason for referral as identified in the present study is even greater. Referral to hospital due to infections in the respiratory system (e.g., pneumonia), urinary tract infections and skin infections (e.g., erysipelas) is reported to be conditions causing inappropriate hospitalizations, and for which interventions in primary care should prevent such [11, 18]. Diffusion of community care programs and services that aim to strengthen both patients and health care personnel on how to observe early signs of clinical and functional decline on a systematic basis is one potential strategy to reduce hospital use among older persons. In this regard, the use of assistive living technologies can have a potential positive impact, as the aim of such interventions is to both strengthen the self-management of chronic diseases, and for health care personnel to use various sensors and monitors to track changes in a patient’s health and vital signs [32, 45]. Lewin and colleagues [33] expect to see a shift from alarm-based telecare systems to systems including more continuous life style monitoring over the next years. This will release a potential for more vigilant and precise follow-up of patients, but the ethics and safety concerning such comprehensive monitoring of persons are a concerns which many stakeholders are addressing now.

In our study cohort, there were substantially fewer men (33%) then women, but men still had a higher annual admission rate; men had an annual admission rate of 1.1, compared to women who had a rate of .9. The mean hospital admission rate for the entire study population was .95. This finding is in accordance with official Norwegian statistics and previous research [21, 22]. However, a study which focused on reduction of inappropriate hospital use, based on analysis of the causes, found no significant differences when comparing the results of inappropriate admission by gender (male/female) [46]. In our study, we found that male gender was a predictor for hospitalizations due to infections, but were not associated with hospitalizations related to falls or general decline. This is supported by a strand of research literature which suggest that men are generally physically stronger and report fewer diseases and have lower levels of primary care use, but higher hospitalization rates and have higher mortality at all ages compared with women: the so-called male-female health-survival paradox [23, 47]. This may suggest that men perhaps disregard early signs of disease and postpone going to the doctor until the later stages of disease development, thus health care personnel in community care must be especially aware of men in the context of prevention of hospitalizations [21].

In our study, we found that age was a predictor for hospitalizations due to general decline, but were not associated with hospitalizations related to falls or infections. This is harmonized with a common understanding that the most problematic expression of an ageing population is the clinical condition of frailty [26]. For this population, it is of vital importance to apply a systematic approach in community care, in order to reduce the use of inappropriate procedures, iatrogenic diseases and nosocomial infections, which are associated with hospitalization [29, 48].

Urgent and emergency services have been the subject of a wide range of policy discourse and decisions over the years, all over Europe. In general, socio-demographic (i.e., age, social deprivation, levels of morbidity, area of residence) factors are associated with increased rates of admissions [18]. These are factors which are highly relevant in understanding other reasons than the clinical conditions for hospitalizations, but in terms of potentially preventing an admission to hospital for the individual patient, it is paramount that personnel in community care are vigilant observers and good clinical practitioners. Proper treatment and care for the most vulnerable, with a view to managing their conditions at home and/or supported by community care, can potentially reduce the risk of hospitalizations, but it also implies to shift resources from hospitals to the community setting, thus reducing the disruptive impact of acute unscheduled hospital admissions [9]. Our study have identified some clinically relevant factors that are vital in this context.

## Limitations

We should mention a number of limitations of the present study. First, we cannot draw any gender-specific conclusion in the present study, due to heterogeneity among populations. Second, diseases with no treatment and asymptomatic conditions could be missed by doctors when recording a medical history, as well as the raters in this study. Third, the findings in this study pertain to the studied municipality in Norway, thus limiting the generalizations of the findings, as financing and organization of health care in Norway is different compared to other countries.

## Conclusions

The potential for preventing hospitalizations for home-dwelling elderly receiving community care lies in discovering and addressing the patients’ symptoms so early that they don’t come to a severe state of illness that requires hospitalization. The most common reasons for referral to hospital were the need for further medical assessment, based on symptoms related to general decline, such as unspecified dyspnea/dehydration/anemia, and referrals related to infections and falls. Our study shows that men are especially at risk for hospitalization with increasing age. This information is vital when vigilant health care personnel in community care make timely and accurate observations. The appliance of assistive living technologies in this context can have a positive impact, as they can be used to track changes in the patients’ vital signs and health condition, but further investigation is needed in this regard.

## Abbreviations

COPD: Chronic Obstructive Pulmonary Disorder
ICD-10: International Classification of Diseases 10th revision
LoS: Length of stay
UN: United Nations
